# Insights Into the Regulation of Offspring Growth by Maternally Derived Ghrelin

**DOI:** 10.3389/fendo.2022.852636

**Published:** 2022-02-18

**Authors:** Takahiro Sato, Takanori Ida, Yuki Shiimura, Kazuma Matsui, Kanae Oishi, Masayasu Kojima

**Affiliations:** ^1^ Division of Molecular Genetics, Institute of Life Science, Kurume University, Kurume, Japan; ^2^ Division for Identification and Analysis of Bioactive Peptides, Department of Bioactive Peptides, Frontier Science Research Center, University of Miyazaki, Miyazaki, Japan; ^3^ Department of Cell Biology, Graduate School of Medicine, Kyoto University, Kyoto, Japan; ^4^ Department of Molecular and Cellular Physiology, Stanford University School of Medicine, Stanford, CA, United States

**Keywords:** offspring, growth, maternal ghrelin, GHS-R, placenta

## Abstract

The regulation of fetal development by bioactive substances such as hormones and neuropeptides derived from the gestational mother is considered to be essential for the development of the fetus. On the other hand, it has been suggested that changes in the physiological state of the pregnant mother due to various factors may alter the secretion of these bioactive substances and induce metabolic changes in the offspring, such as obesity, overeating, and inflammation, thereby affecting postnatal growth and health. However, our knowledge of how gestational maternal bioactive substances modulate offspring physiology remains fragmented and lacks a systematic understanding. In this mini-review, we focus on ghrelin, which regulates growth and energy metabolism, to advance our understanding of the mechanisms by which maternally derived ghrelin regulates the growth and health of the offspring. Understanding the regulation of offspring growth by maternally-derived ghrelin is expected to clarify the fetal onset of metabolic abnormalities and lead to a better understanding of lifelong health in the next generation of offspring.

## Introduction

Ghrelin is a peptide hormone purified from the stomach as an endogenous ligand for the GH secretagogue receptor (GHS-R) (1). The ghrelin receptor GHS-R is highly conserved from fish to humans and is widely expressed in central and peripheral organs such as the brain, pituitary gland, and pancreas (2–7). Therefore, the ghrelin-GHS-R system is involved in the regulation of various physiological functions such as growth hormone (GH) secretion (1), feeding (8), body temperature (9), gastric motility (10), gastric acid secretion (11), insulin and gastrin secretion (12), circulatory systems (13, 14), and stress responses (14). Neonatal rats treated with ghrelin showed faster eye and vaginal opening, indicating that ghrelin is also involved in neonatal development (15). Furthermore, in rodents, it has been shown that ghrelin secreted from the stomach and placenta of pregnant mothers may also act on the fetus (16), suggesting that maternally-derived ghrelin may regulate fetal growth and affect postnatal health. In this mini-review, we will first outline the molecular structure and physiological function of ghrelin to understand the changes in maternal physiological status and ghrelin secretion. Next, we will advance our understanding of the role of maternal ghrelin in the regulation of offspring growth, particularly in the fetus, and discuss the effects of maternal ghrelin on the maintenance and disruption of offspring health after birth.

## Basic Knowledge About Ghrelin and GHS-R

### Molecular Structure and Distribution of Ghrelin

**Figure 1:**
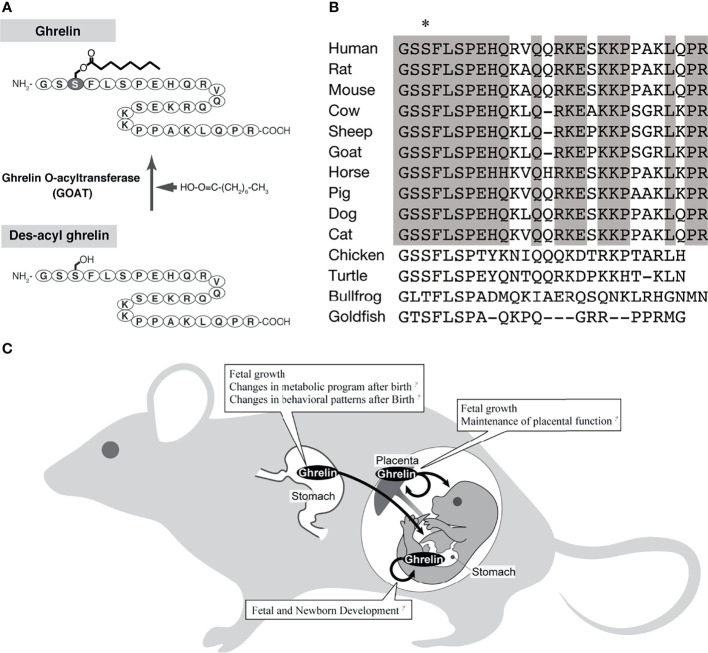
The biochemistry of ghrelin and the action of ghrelin in the fetus. **(A)** Biosynthesis of ghrelin. The active form of ghrelin is produced by the action of a ghrelin-specific fatty acid modifying enzyme (GOAT). **(B)** Comparison of ghrelin sequences, mainly from mammals (modified from Ida et al., 2010) (17). Asterisk indicates amino acid residues with fatty acid modifications. Gray boxes indicate amino acids that are highly conserved among mammals. **(C)** A schematic diagram of the pathway of ghrelin from the mother to the fetus.

Ghrelin is a peptide hormone discovered in rat and human stomachs as an endogenous ligand for GHS-R (1) (**Figure 1A**). Human ghrelin is a bioactive peptide of 28 amino acid residues with a molecular weight of 3,370.9. The side chain of the third serine residue is esterified with octanoic acid (molecular weight 144), a fatty acid with an 8-carbon chain containing no double bond (1). Ghrelin has been identified in a variety of mammals, birds, reptiles, amphibians, and fish, all of which have the third serine or threonine residue modified by a fatty acid (1, 18–26) (**Figure 1B**). Ghrelin is a potent stimulator of GH secretion both *in vitro* and *in vivo*, hence the name “ghrelin” is based on the Indo-European languages “ghre” for “grow” (1). The name “ghrelin” also includes the meaning “to release” GH (1). On the other hand, ghrelin molecules that are not modified with fatty acids are called desacyl ghrelin and do not have GH release activity (27). Ghrelin is the only peptide hormone in mammals that is acylated and modified by octanoic acid, and this acylation modification is catalyzed by ghrelin O-acyltransferase (GOAT) (28) (**Figure 1A**). When conducting research on ghrelin, it is necessary to consider the characteristic molecular structure of ghrelin and the associated biosynthetic pathway.

Ghrelin is most abundant in the stomach and is also secreted by the duodenum (26, 27, 29–33). Other small amounts of ghrelin production have been found in the hypothalamus, pancreas, kidney, and placenta (5, 34–37) In the stomach, ghrelin is most abundant in the gastric fundus where it is produced in oxyntic glands, and is a closed endocrine cell not in contact with the lumen (29). Ghrelin-producing cells have been called X/A-like cells because they contain many secretory granules and resemble A cells that produce glucagon in the pancreas (26, 29, 38). Ghrelin has been reported to be stored in electron-dense secretory granules of nearly uniform size, 120 nm in diameter. Ghrelin cells account for 20-25% of the endocrine cells in the gastric body and are the second most abundant endocrine cells after histamine-producing enterochromaffin-like cells (29).

### Molecular Structure and Distribution of GHS-R

The GHS-R gene consists of two exons: the first exon encodes the first through fifth transmembrane regions, and the second exon encodes the sixth through seventh transmembrane regions (39, 40). The ghrelin receptor gene produces two types of mRNAs, type 1a and type 1b. Type 1a receptor binds ghrelin as a seven-transmembrane G protein-coupled receptor (GPCR), whereas type 1b is a five-transmembrane receptor and does not bind ghrelin (40). Recently, several ghrelin receptor structures have been determined, including the active-ghrelin binding form (41–43). The ligand-binding pocket of the ghrelin receptor has a unique architecture, a bifurcated pocket not found in closely related GPCRs, and this structural feature is known to be utilized to recognize the octanoic acid modification of ghrelin. After activation, GHS-R binds to the trimeric Gq protein and promotes Ca^2+^ release from the endoplasmic reticulum *via* activation of phospholipases and production of inositol 3-phosphate (40). This Ca^2+^ pathway is the intracellular signaling system of ghrelin.

GHS-Rs are highly conserved from fish to humans and are widely expressed in central and peripheral organs such as the brain (hypothalamus, cerebral cortex, hippocampus, substantia nigra, brainstem, etc.), pituitary gland, and pancreas (2–7). This broad distribution of GHS-R allows ghrelin to exhibit a wide variety of physiological effects.

### Physiological Functions of Ghrelin

Ghrelin has a wide variety of physiological effects that can affect maternal maintenance and fetal growth. In particular, the stimulation of GH release from the pituitary gland and the enhancement of feeding in the hypothalamus are known to be the major physiological effects of the ghrelin/GHS-R axis.

#### Regulation of Growth Hormone Secretion by Ghrelin

Although many factors regulate the synthesis and secretion of GH in the pituitary gland, GH secretion is essentially regulated by three ligand/ligand-receptor axes the classically known growth hormone-releasing hormone (GHRH)/GHRH receptors of the hypothalamic-pituitary axis, somatostatin (SST)/SST receptors, and ghrelin/GHS-R. In terms of GH secretion, GHRH/GHRH receptors are primarily responsible for GH synthesis and secondary to GH release, ghrelin/GHS-R are primarily responsible for GH release and secondary to GH synthesis, and somatostatin/somatostatin receptors work to inhibit GH release (1, 44). Ghrelin secreted from the stomach stimulates GH secretion by stimulating afferent vagal nerves expressing nearby GHS-Rs (45). Ghrelin secreted from ghrelin neurons in the hypothalamic arcuate nucleus also binds to GHS-Rs expressed on GHRH neurons to release GHRH and induce GH secretion (46).

#### Hyperphagic Effect of Ghrelin

Another major physiological effect of ghrelin is its ability to promote food intake. When ghrelin is administered centrally or peripherally to rats or mice, it promotes feeding and weight gain (8, 47, 48). However, ghrelin-induced food intake is not associated with GH secretion. In addition, subcutaneous transplantation of a ghrelin-producing cell line into nude mice increases blood ghrelin levels, increasing food intake (49). This cell line maintains the physiological regulation of ghrelin secretion, such that ghrelin secretion is decreased by the addition of glucose or insulin. Therefore, the blood ghrelin level of nude mice transplanted with this cell line is thought to have increased while retaining the physiological ghrelin secretion pattern to some extent.

Orexigenic peptides such as neuropeptide Y (NPY) and agouti-related peptide (AgRP) are present in the hypothalamus. These peptides exhibit an orexigenic effect when administered intraventricularly but not when administered peripherally. The orexigenic effect of ghrelin occurs not only by intracerebro-ventricular administration but also by intravenous and intraperitoneal administration, indicating that ghrelin is the only peripheral hunger signal (8). The hypothalamus contains an important central nucleus involved in the regulation of food intake, where a large amount of information is integrated to control energy metabolism. The orexigenic effect of ghrelin occurs when ghrelin activates NPY/AgRP neurons expressing GHS-R and promotes the secretion of NPY and AgRP (8). Ghrelin administered intravenously also activates NPY/AgRP neurons to promote feeding (45). The involvement of the vagus nerve, a brain nerve that transmits various information from the gastrointestinal tract to the interneurons and neocortex *via* the brainstem, has been reported as a pathway for the central effects of ghrelin secreted from the stomach (45). According to this report, ghrelin binds to GHS-R, which is produced by vagal afferent neurons and transported to afferent fiber terminals, and inhibits the electrical activity of vagal afferent fibers. In addition, ghrelin information is transmitted to the solitary nucleus of the medulla oblongata, where it changes neurons and stimulates NPY/AgRP neurons in the hypothalamus, thereby promoting feeding. However, since there are some reports on the pathway through the blood-brain barrier (50–52), it is necessary to continue to monitor the progress of research on how ghrelin secreted from the stomach acts on the central nervous system.

#### Regulation of Energy Metabolism

Ghrelin is also involved in the regulation of energy metabolism. Continuous subcutaneous administration of large amounts of ghrelin increases body weight, and since the respiratory quotient increases at this time, it is thought that this is due to an increase in adipose tissue associated with suppression of body fat utilization (47). In mice, ghrelin maintains the diurnal rhythm of body temperature and induces torpor, an energy-conserving phenomenon in which body temperature is markedly lowered during starvation (9). In humans, plasma ghrelin concentration is inversely correlated with body mass index (BMI), and is reported to be low in obese individuals and high in cases of anorexia nervosa, severe heart failure, and lung cancer with strong cachexia. This suggests that ghrelin is activated during negative energy balance and maintains homeostasis by stimulating food intake, fat accumulation, and lowering body temperature. Thus, ghrelin regulates energy metabolism through a variety of functions, but it has been reported that there is no correlation between ghrelin levels and gestational weight gain in overweight and normal groups in pregnant humans.

#### Effects of Ghrelin On the Cardiovascular System

Ghrelin also acts in the regulation of the cardiovascular system. When ghrelin is administered continuously to a rat model of chronic heart failure, improvements in cardiac function are observed, including a decrease in peripheral vascular resistance, an increase in cardiac output, an increase in left ventricular ejection fraction, inhibition of left ventricular remodeling development, and promotion of compensatory cardiac hypertrophy in the non-infarcted area (14). This is thought to be a direct effect of ghrelin and an effect mediated by GH/insulin-like growth factor-1 (IGF-1), which is increased by ghrelin (14). Ghrelin also causes increased blood flow and decreased blood pressure in a GH- and IGF-1-independent manner, which may be due to the vasodilator effect of ghrelin, since GHS-R is also present in blood vessels (53). In addition, a single dose of ghrelin intravenously in healthy subjects has a relatively long-lasting blood pressure (mean arterial pressure) lowering effect of about 10 mmHg, along with an increase in plasma GH concentration, and also increases cardiac index and cardiac output without changing heart rate (13). This indicates that ghrelin alters circulatory dynamics by suppressing the sympathetic nervous system. As mentioned above, plasma GH and ghrelin concentrations are elevated in patients with chronic heart failure associated with cachexia, but they are positively correlated with BMI, plasma tumor necrosis factor-α, and plasma GH concentrations (54), suggesting that ghrelin functions in a compensatory manner in the pathogenesis of cachexia, in which energy metabolism tends toward catabolism. In preeclampsia, a known complication of pregnancy, ghrelin secretion is increased, and serum ghrelin levels have been reported to correlate negatively with blood pressure (55, 56). It has been suggested that this is because ghrelin improves endothelial function by enhancing angiogenesis through the Jagged1/Notch2/VEGF pathway (57).

## Maternal Ghrelin and Fetal Growth

Various perspectives on the effects of ghrelin on fetal growth have been reported (**Figure 1C**), including ghrelin in the maternal circulating blood acting through the placenta and by placenta-derived ghrelin (**Table 1**). In the rat fetus, ghrelin mRNA has been slightly observed in the stomach from late pregnancy, whereas GHS-R mRNA has been detected at high levels in various peripheral fetal tissues from as early as embryonic day 14, and has also been confirmed by immunohistochemistry (16, 59, 61). Therefore, the regulation of fetal growth by ghrelin is thought to be essentially due to maternally derived ghrelin.

**Table 1:** Detection of ghrelin in the placenta.

Animal species	Detection Sites	Detection Periods	Detection Methods	References
**Human**				
Ghrelin	Cytotrophoblast cells.	First-trimester.	Immunohistochemistry	(36)
Ghrelin mRNA		First trimester and after delivery.	RT-PCR	(36)
**Rat**				
Ghrelin	The cytoplasm of labyrinth trophoblast.	Day 21 of pregnancy.	Immunohistochemistry	(36)
Ghrelin mRNA		Almost undetectable during early pregnancy, with a sharp peak of expression at day 16 and decreasing in the latest stages of gestation. Embryonic day 17 (E17).	RT-PCR & Northern blot *In situ* hybridization	(36) (58)
**Mouse**				
Ghrelin	Labyrinthine trophoblast cells	Gestational day 17.5 (GD17.5).	Immunohistochemistry	(59)
**Ovine**				
Ghrelin	The maternal epithelium, caruncle and trophectoderm.	All gestational time points.	Immunohistochemistry	(60)

Maternal ghrelin includes ghrelin derived from the placenta and ghrelin derived from the maternal circulating blood (36, 58–60, 62). Placental ghrelin has been found in human and rat placentas, and ghrelin mRNA has been detected both in the first trimester and postpartum in humans (36). Immunohistochemical analysis has also shown that ghrelin is expressed in the human placenta in the first trimester in cytotrophoblasts and rarely in syncytiotrophoblasts (36). In addition, ghrelin was also identified in BeWo cells, a cell line of human choriocarcinoma (36). Ghrelin is also detected by immunohistochemistry in the cytoplasm of labyrinth trophoblast of the rat placenta, but placental ghrelin mRNA from pregnant rats shows a characteristic expression profile in which ghrelin is almost undetectable in early pregnancy, reaches its peak expression on day 16 of pregnancy, and decreases in late pregnancy (36). In other words, ghrelin is present in both human and rat placentas and shows a pregnancy-related time course of expression (36). It has been suggested that the function of placental-derived ghrelin may be to influence postnatal weight gain, but this appears to be limited to cases where the mother’s weight is normal (62).

On the other hand, research on ghrelin derived from maternal circulating blood was been conducted early in the discovery of ghrelin. It was found that a single injection of ghrelin into the mother increased the concentration of circulating ghrelin in the fetus within 5 minutes after injection, and it was reported that maternal ghrelin was readily transferred to the fetal circulation (16). This study also found that chronic treatment of rat mothers with ghrelin increased birth weight, and that restricting maternal food intake with paired feeding after ghrelin administration stimulated fetal growth, while active maternal immunization decreased fetal weight during pregnancy (16). It has also been reported that maternal ghrelin administration can alter the behavior of the offspring (15). Administration of ghrelin to pregnant mice suppressed exploratory behavior of the pups in an open field test (15). The pups had high plasma levels of basal corticotropin-releasing hormone and did not respond to acute restraint stress (15). It has also been found that repeated maternal restraint stress increases maternal and fetal plasma acyl ghrelin concentrations (15). Thus, it can be seen that increased maternal ghrelin under physiological conditions such as stress is transported to the fetus and alters the endocrine environment of the offspring, affecting their behavior. In addition, female wild-type mice born from ghrelin heterozygous dams exposed to ghrelin deficiency *in utero* have reduced reproductive capacity and smaller litters (63). In addition, implantation of wild-type embryos into the uterus of mice exposed to intrauterine ghrelin deficiency results in a 60% reduction in implantation rate compared to embryos implanted in non-exposed uteri (63). Uterine expression of several genes important for implantation is also altered in the uterus of mice exposed to intrauterine ghrelin deficiency, and abnormalities in endometrial proliferation have been observed (63), leading to the conclusion that exposure to reduced ghrelin in the uterus causes defects in uterine developmental programming and subsequent infertility in wild-type offspring.

Thus, we can see that ghrelin derived from the placenta and maternal circulating blood maintains pregnancy by regulating implantation and fetal growth, and also influences the endocrine environment and behavior of the offspring after birth. However, since studies with ghrelin gene-deficient mice have not found abnormalities in the number of pups or newborn weight (9, 64), it is essential to understand the role of maternally derived ghrelin while clarifying the effects of endogenous and exogenous ghrelin.

## Future Perspectives

As mentioned in the introduction, the regulation of fetal growth by bioactive substances such as hormones and neuropeptides derived from the gestational mother is essential. However, there remains a lack of knowledge on how the modulation of these bioactive substances leads to future health and susceptibility to certain diseases. To accumulate such knowledge, it is necessary to promote research on bioactive substances of gestational maternal origin, whose secretory dynamics have been elucidated, which have also been shown to act reliably on the fetus, and whose involvement in disease has been clarified. In this regard, ghrelin may be one of the most important targets for understanding the fetal onset of metabolic abnormalities.

Although ghrelin has a variety of physiological effects, it is comprehensively a hormone that maintains homeostasis by exerting anabolic effects in hyper-catabolic conditions such as fasting, insulin, and leptin administration. Ghrelin secretion is known to be altered by ingesting factors that may be related to lifestyle, such as a high-fat diet or nicotine (47, 65), and is elevated in hyperemesis gravidarum, a physiological condition unique to pregnant women (66). In addition to these reports, the fact that ghrelin is secreted by or passes through the placenta in pregnant females implies that changes in ghrelin secretion due to maternal preferences, lifestyle, and physiological conditions may affect the health of the offspring in various ways. Furthermore, it has been suggested that the hyperghrelinemia during maternal undernourishment rewires the hypothalamic development of the offspring when fed a high-fat diet, affecting GHS-R signaling and contributing to the hyperphagia and the increase in body weight (67). Although this report does not describe the effects of maternally-derived ghrelin, it shows that maternal malnutrition also affects GHS-R signaling in the offspring, which is an important finding when considering offspring growth.

As the world’s population is currently increasing, the elderly population is also expected to increase along with the development of medical science. Therefore, it is necessary to identify high-risk groups at prenatal and developmental stages, and to realize preemptive medicine through early intervention using food components and other means. Ghrelin is a peptide hormone with a characteristic molecular structure, and it is known that octanoic acid, a side chain necessary for the expression of activity, is added simply by oral administration (68). This suggests that ghrelin may be a good target molecule for healthy longevity.

## Conclusion

There are two main types of maternally-derived ghrelin: ghrelin secreted from the placenta and ghrelin secreted from the stomach and passed through the placenta. The mechanisms by which these ghrelin act during the embryonic period of the offspring are still under investigation, but as described in this mini-review, maternally-derived ghrelin appears to regulate implantation and fetal growth, maintain pregnancy, and even influence the endocrine environment and behavior of the offspring after birth. Therefore, abnormal maternal ghrelin secretion may interfere with the normal growth of the offspring during embryonic and developmental stages, leading to subsequent functional disorders and diseases. In the future, it is hoped that the mechanisms by which maternally-derived ghrelin regulates fetal function will be elucidated at the molecular level, and that this knowledge will be coupled with an understanding of healthy longevity.

## Author Contributions

TS examined the data and wrote the manuscript. TS, TI, YS, KM, KO, and MK contributed to the discussion and reviewed the manuscript. All authors contributed to the article and approved the submitted version.

## Funding

This work was funded by a Grant-in-Aid for Scientific Research (C) (No.20K08898, to TS) from JSPS KAKENHI; and grants (to TS) from the Ishibashi Foundation for the Promotion of Science, the Takeda Scientific Foundation, and the Kobayashi Foundation (No. 215) in Japan.

## Conflict of Interest

The authors declare that the research was conducted in the absence of any commercial or financial relationships that could be construed as a potential conflict of interest.

## Publisher’s Note

All claims expressed in this article are solely those of the authors and do not necessarily represent those of their affiliated organizations, or those of the publisher, the editors and the reviewers. Any product that may be evaluated in this article, or claim that may be made by its manufacturer, is not guaranteed or endorsed by the publisher.
